# Overview on the Prevalence of Fungal Infections, Immune Response, and Microbiome Role in COVID-19 Patients

**DOI:** 10.3390/jof7090720

**Published:** 2021-09-02

**Authors:** Maryam Roudbary, Sunil Kumar, Awanish Kumar, Lucia Černáková, Fatemeh Nikoomanesh, Célia F. Rodrigues

**Affiliations:** 1 Department of Parasitology and Mycology, School of Medicine, Iran University of Medical Sciences, Tehran 1449614535, Iran; roudbari.mr@iums.ac.ir; 2 Faculty of Biosciences, Institute of Biosciences and Technology, Shri Ramswaroop Memorial University, Barabanki 225003, Uttar Pradesh, India; sunil.bio@srmu.ac.in; 3 Department of Biotechnology, National Institute of Technology, Raipur 492010, Chhattisgarh, India; 4 Department of Microbiology and Virology, Faculty of Natural Sciences, Comenius University in Bratislava, Ilkovičova 6, 842 15 Bratislava, Slovakia; lucia.cernakova@uniba.sk; 5 Infectious Disease Research Center, Birjand University of Medical Sciences, Birjand 9717853577, Iran; g.nikoomanesh@yahoo.com; 6 LEPABE—Laboratory for Process Engineering, Environment, Biotechnology and Energy, Faculty of Engineering, University of Porto, Rua Dr. Roberto Frias, 4200-465 Porto, Portugal

**Keywords:** fungal infection, COVID-19, SARS-CoV-2, immune response, *Candida*, Aspergillus, *Mucor*, immune response, microbiome

## Abstract

Patients with severe COVID-19, such as individuals in intensive care units (ICU), are exceptionally susceptible to bacterial and fungal infections. The most prevalent fungal infections are aspergillosis and candidemia. Nonetheless, other fungal species (for instance, *Histoplasma* spp., *Rhizopus* spp., *Mucor* spp., *Cryptococcus* spp.) have recently been increasingly linked to opportunistic fungal diseases in COVID-19 patients. These fungal co-infections are described with rising incidence, severe illness, and death that is associated with host immune response. Awareness of the high risks of the occurrence of fungal co-infections is crucial to downgrade any arrear in diagnosis and treatment to support the prevention of severe illness and death directly related to these infections. This review analyses the fungal infections, treatments, outcome, and immune response, considering the possible role of the microbiome in these patients. The search was performed in Medline (PubMed), using the words “fungal infections COVID-19”, between 2020–2021.

## 1. Introduction

**Figure 1:**
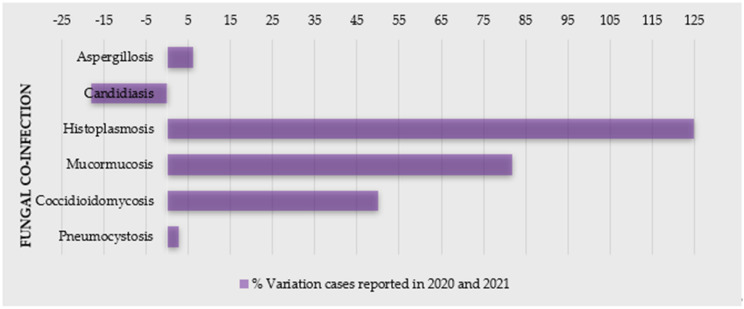
Percentage of variation of cases of COVID-19 patients with fungal co-infections reported in 2020 and 2021 (source: PubMed).

Severe acute respiratory syndrome coronavirus 2 (SARS-CoV-2), the etiologic agent of coronavirus disease 2019 (COVID-19), has infected millions of patients worldwide, and placed an unprecedented stress on healthcare systems [1,2,3,4]. This disease has predisposed a relatively high number of patients to acute respiratory distress syndrome, and co-infections are a frequent complication [5,6], especially with prolonged hospital stays [7]. Changes in humans’ microbiota have been recently observed in COVID-19 patients [1], with patients often being colonized or infected by microorganisms responsible for secondary infections (co-infections or superinfections), often caused by bacteria and fungal pathogens [5,7,8,9]. Indeed, several opportunistic infections following severe respiratory viral infections have been recognized in COVID-19 patients [2]—particularly, a higher incidence of fungal co-infections (Figure 1) [10,11,12]. For example, in Spain, the incidence of candidemia cases was higher in the first and second waves and lower during the third wave, also with a prevalence of invasive pulmonary aspergillosis (IPA) cases [11]. Moreover, the coronavirus-associated pulmonary aspergillosis (CAPA) showed to affect up to 30% of ventilated patients with COVID-19 admitted in intensive care units (ICU) [13], and, in a hospital in Pisa (Italy), 21.9% of 315 hospitalized patients with COVID-19 had a superinfection [14].

The main pathogens related to co-infections are reported to be Enterobacterales (44.9%), non-fermenting Gram-negative bacilli (15.6%), Gram-positive bacteria (15.6%), and fungi (5.5%) [14]. In COVID-19 patients, the most fungi related to co-infections are *Aspergillus* spp., *Candida albicans, Candida glabrata*, *Candida dubliniensis*, *Candida parapsilosis sensu stricto*, *Candida tropicalis*, and *Candida krusei (Pichia kudriavzevii)* [8]. Moreover, these cases have been indicated as mainly primary and catheter-related infections [15].

There is still lack of information regarding the long-term impact of secondary infections on the outcome of hospitalized COVID-19 patients [9,16]. Patients with co-infection undergoing invasive mechanical ventilation showed to be 3.8 times more likely to die than those without positive cultures [9]. In order to perform an efficient treatment and reduce mortality, it is important to make an accurate early identification [12]; however, these co-infections raise difficulties on diagnosis, treatment (including broad-spectrum antimicrobial drugs, mechanical ventilation, extracorporeal membrane oxygenation), prognosis, and even increase the disease the symptoms and mortality of COVID-19 [8,12,15,17,18,19].

The repercussions of SARS-CoV-2 infections on future global antimicrobial resistance must be explored profoundly [3,16]. In Valencia (Spain), the antifungal consumption increased in 2020 compared to previous year, especially echinocandins, voriconazole, and isavuconazole [11]. Considering that the antimicrobials drugs for COVID-19 patients, both on and during admission, are almost all prescribed uncertainly in clinical settings, there is expected an increase in drug-resistant infections [3].

Lastly, considering the immune response, there has been represented a host dysregulation triggered by SARS-CoV-2 infection, which has been hypothesized as a causal pathway for the increasingly reported mainly fungal (oral) manifestations associated with COVID-19 [20,21]. Additionally, the alteration in human microbiota (due to SARS-CoV-2 infection), which can also indicate the progression of COVID-19, may contribute to bacterial, fungal, or viral infections and affect the immune system [1]. In these patients, this is normally described as an increase in pro-inflammatory markers, such as IL-1, IL-6, and tumor necrosis alpha (TNF-α), less CD4 interferon-gamma expression, and a decreased number of CD4 and CD8 cells, which increase susceptibility to bacterial and fungal infections [12].

The present review aims to analyze the prevalence of fungal infections, immune response, and the role of the microbiome in COVID-19 patients.

## 2. Fungal Infections as a Co-Morbidity of COVID-19

Fungal co-infections are frequent in the COVID-19 patients; therefore, its awareness is important for proper diagnosis and, subsequently, efficient treatment of the fungal co-infections for reducing morbidity and mortality. Due to a general neglected approach towards fungal tropical diseases, morbidity and mortality is expected to worsen in the context of the COVID-19 pandemic [22]. SARS related to COVID-19 disease is known to increase the risk of invasive fungal infections (IFI) [23,24]. In addition, patients suffering from endemic mycoses and COVID-19 co-infection seem to be an at-risk population and have a poor prognosis. A significant number of cases of COVID-19-associated candidiasis, aspergillosis, mucormycosis, and histoplasmosis have been reported so far from the different region of the world [22,25,26,27]. Some reports even state that COVID-19 increases the mortality rate in the patients having fungal infections, but the case reports suggest that individuals with COVID-19 are more susceptible to a fungal infection mostly because of impaired immune responses, which further increases the awareness of clinicians for more effective diagnosis and treatment [28,29].

### 2.1. Candidiasis

One of the major complications of severe COVID-19 cases are yeast infections. They are mainly caused primarily by *Candida* spp., which are associated with a high mortality rate, due to a longer ICU stay, catheterization, and broad-spectrum antibiotic use [6] (Table 1). Nucci et al. observed stable incidence of candidemia in their hospital during an 18-year period (1.3 episodes per 1000 admissions), but since March 2020, an increase in cases diagnosed with candidemia was noticed [30]. Compared with non-COVID-19 patients, COVID-19 patients with candidemia were more likely to be under mechanical ventilation [30]. Katz et al. evaluated the association between COVID-19 and oral and systemic candidiasis [25]. Generally, candidiasis was significantly associated with increased risk for COVID-19, whereas oral candidiasis showed an insignificant trend [25].

Both fungi and virus display highly distinctive patterns of sudden emergence, and are based on simple infection-driven, human-to-human transmission [31]. In times of SARS-CoV-2, the vigilance of multidrug-resistant *Candida* spp. (e.g., *Candida auris*, *C. glabrata*, and *Candida duobushaemulonii* [17,32,33]) is extremely important. Data regarding multidrug-resistant *Candida* spp. in COVID-19 patients are scarce [32]. *C. auris*, an emerging pathogen known for a reduced susceptibility to antifungals, is spread across all continents [5], and it is easily transmitted between healthcare professionals. Both *C. auris* and SARS-CoV-2 have been found on hospital surfaces including on bedrails, intravenous (IV) poles, beds, air conditioner ducts, windows, and hospital floors [5]. Hospital-acquired *C. auris* infections in coronavirus disease patients may lead to adverse outcomes and additional strain on healthcare resources [34]. Moreover, the standard COVID-19 critical care of using mechanical ventilation and protracted ventilator-assisted management makes these patients potentially susceptible to colonization and infections by *C. auris* [5]. For example, during April–July 2020 in New Delhi (India), *C. auris* accounted for two-thirds of cases, and the case-fatality rate was very high (60%) [4]. In a phylogenetic molecular clock study (Genoa, Italy), Di Pilato and colleagues showed that all *C. auris* isolates were resistant to amphotericin B, voriconazole, and fluconazole at a high level, owing to mutations in *ERG11* (K143R) and *TACB1* (A640V) genes. Critically, *C. auris* could be easily spread because of the COVID-19 pandemic [35]. After the first *C. auris*-colonized case was diagnosed in a COVID-19 patient in ICU at a hospital in Salvador, Brazil, a multidisciplinary team conducted a local *C. auris* prevalence investigation [36]. Remarkably, findings revealed that among body swabs collected from 47 patients, eight samples from the axillae were positive for *C. auris*. Contaminated axillary monitoring thermometer helped to *C. auris* dissemination. Re-use of these devices must imply a careful disinfection or they should be replaced by other temperature monitoring methods [36]. Moreover, in 2020, the Florida Department of Health was alerted to three *C. auris* bloodstream infections and one urinary tract infection (UTI) in four patients with COVID-19 who had received care in the same COVID-19 ICU ward [37]. A report from in a tertiary academic center (United States, May 2014 to October 2020) showed that in an entire sample (non-COVID-19 and COVID-19 groups), *C. albicans* accounted for a minority of isolates collected [38]. Compared to non-COVID-19 patients with candidemia, COVID-19 patients had lower ICU admission sequential organ failure assessment score, but longer ICU stays and central venous catheter dwell times at candidemia detection [38].

Surveillance data assessed differences in candidemia patients with and without a prior COVID-19 diagnosis [28]. COVID-19 patients with candidemia lacked established underlying conditions associated with candidemia but had two times the mortality rate versus candidemia patients without COVID-19 [28]. Over a two-year period, patients followed in the ICU of Ankara City Hospital, Turkey, were divided into pre-pandemic and pandemic periods [29]. In multivariate logistic regression analysis, corticosteroid use, presence of sepsis, and age older than 65 years were independent risk factors for mortality in candidemia patients [29]. Indeed, candidemia with high mortality is reported as a more serious problem for COVID-19 patients due to its increased and earlier incidence, and a higher rate of mortality [28,29].

### 2.2. Aspergillosis

Aspergillosis is one of the most common opportunistic fungal co-infections caused by some *Aspergillus* spp., which particularly affects immunocompromised persons, such as COVID-19 patients. It critically affects the respiratory system, leading to a mild/serious lung infection, known as pulmonary aspergillosis, a serious form of aspergillosis, which becomes worse over time and does not have an effective treatment [26,41]. Clinical characteristics of the COVID-19 patients co-infected with aspergillosis can be analyzed in Table 2. Based on the available literature, it is suggested to keep a low threshold to investigate for COVID-19 associated pulmonary aspergillosis (CAPA), since an early detection and respective treatment may significantly improve outcomes. Moreover, prolonged courses of steroids should not be given unless further conclusive evidence is available [42], because steroids suppress the immune system, making the patient more susceptible to secondary infections. A rapid and aggressive treatment approach with judicious use of steroids while treating co-infections turns out to be the best possible outcome and solution.

### 2.3. Histoplasmosis

Histoplasmosis is a systemic mycosis, highly endemic in certain regions of America and Asia, including Brazil and India. It is caused by a dimorphic fungus, *Histoplasma capsulatum,* which predominately occurs in soil containing large amounts of bird or bat droppings. The infection occurs through the inhalation of fungal microconidia after perturbation of these environmental sources [50]. Similarly to aspergillosis, the disease is usually associated with immunosuppressive conditions, clinically presenting severe acute disseminated forms. Underlying lung disorders can predispose individuals to chronic pulmonary histoplasmosis, whereas acute and subacute pulmonary forms mainly occur in healthy individuals after a large fungal inoculum inhalation [50,51]. These clinical forms are less known, often misdiagnosed as bacterial pneumonia and pulmonary tuberculosis (Table 3). In the case of this particular fungal disease, it was indicated that most patients who received steroids for COVID-19 treatment developed histoplasmosis (Table 3). Histoplasmosis is mainly associated with COVID-19 patients with AIDS, and there are very few studies on the co-infection of *H. capsulatum* and COVID-19 [27,52]. Actually, the important findings were all patients of COVID-19 having co-infection of *H. capsulatum* survived after antifungal treatment with amphotericin B and itraconazole (Table 3) [27,52,53,54,55].

### 2.4. Mucormycosis

The presence of hyphal infiltration of sinus tissue and a temporal course of less than four weeks defines mucormycosis [56,57]. The most common species related to mucormycosis are *Rhizopus* spp. and *Mucor* spp., but recently, a new *Cunninghamella* species, *Cunninghamella* bigelovii, was described [58]. Clinically, rhino-cerebral mucormycosis (RCM) can have atypical symptoms and signs that are similar to complicated sinusitis, such as crusting, nasal blockage, facial pain, proptosis and chemosis, edema, ptosis, and even ophthalmoplegia, as well as fever and headache and symptoms of intracranial extension [59,60]. A black eschar can be found on the hard palate or in the nasal cavity, but it is not typical [61,62]. Mycotic infiltration of blood vessels, thrombosis with vasculitis, acute neutrophilic infiltrate, bleeding, and tissue infarction are all histological characteristics [63].

Without early treatment and identification, this illness may advance quickly, with reported death rates of 50–80%, due to intra-orbital and cerebral complications. Even with timely treatment of underlying illnesses, diagnosis, and surgical intervention, therapy is frequently ineffective, resulting in infection spread and eventually death [64].

Recently, there has been a shift in the occurrence of sinus mucormycosis infection, and patients have been identified more often. A dramatic increase in cases of invasive fungal sinusitis, especially mucormycosis, has occurred in the past months, with many patients needing drastic surgical operations to treat this illness [65,66]. The use of steroids to control COVID-19 may be directly related to the suppression in immunity; thus, it also allows the colonization of opportunistic fungi, leading to mucormycosis, during any stages of the disease (Table 4) [23].

### 2.5. Cryptococcus

*Cryptococcus neoformans* is also related to a very serious opportunistic infection in immunocompromised patients. It has been reported that *C. neoformans* can infect COVID-19 patients. Mohamad Y et al. described the importance of early suspicion of *C. neoformans* infections in patients with immunocompromised state, considering that Cryptococci patients have a high risk of mortality [98]. In the current perspective, the use of immunosuppressive drugs should be justified and to be alert for infections such as *C. neoformans*, which can cause sepsis and mortality [98]. Studies have shown that almost all patients with COVID-19 having co-infection of *C. neoformans* did not survive, even after treatment with fluconazole and amphotericin B (Table 5).

### 2.6. Other Fungal Infections

Some other types of fungal infections have also been reported along with COVID-19. This is the case of *Coccidioides immitis* and *Pneumocystis jirovecii* (Table 5). Although co-infection with *P. jirovecii* is considered life-threatening, according to recent publications, patients improved clinically when treated with common drugs, such as trimethoprim–sulfamethoxazole [99,100]. Similarly to the other cases, during these co-infections, steroids had a negative impact on COVID-19-associated fungal co-infections conditions [100,101].

## 3. Role of Immune Response against the Most Clinically Relevant Fungal Infections in COVID-19 Patients: Two Sides of a Coin

The profound role of the host immune system to fight against fungal pathogens has been extensively described. Generally, two mainly types of immune cells, related to innate and adaptive immunity, dynamically contribute to effective immunity to eliminate the fungal pathogens [114,115].

Since COVID-19 patients are immunosuppressed, the adaptive form of immunity (lymphocytopenia in lymphocytes T CD4^+^ and CD8^+^) is remarkably declined, thus having a defective immune response [12]. As such, it is quite reasonable speculate that these factors establish a favorable environment for the acquisition of persistent fungal co-infections [116,117,118]. Moreover, collateral effects of host recognition pathways, which are desirable for the activation of antiviral immunity, may unexpectedly contribute to a highly permissive inflammatory environment. This, of course, favors fungal pathogenesis and predisposes patients to opportunistic fungal infections, with an exceptional chance of inducing the pathogenicity in high-risk patients [119]. In the beginning of the COVID-19 pandemic, the most predominant fungal infections were pulmonary aspergillosis [116] and candidiasis [6]. Recently, mucormycosis and cryptococcosis [12] are also among the main opportunistic fungal infections in vulnerable groups.

It is known that invasive yeast infections (IYF), especially candidiasis, are dramatically rising in COVID-19 patients, causing complications mainly related to oral infections [120] and candidemia [121]. Some reports have indicated that, in spite of impaired immune response of COVID-19, immune cells accounting for immunity in candidiasis are still present. Thus, it is probable that relevant clinical risk factors play critical role in developing IYF on COVID-19 cases [6]. This is the case of wide-spectrum antibiotic/steroid use, prolonged ICU stays and central venous catheters, transplant patients, chemotherapy/radiotherapy, patients under invasive or noninvasive ventilation, and diabetic individuals [6,119].

Changes in immune phenotype and cytokine release by whole blood stimulation assays against *A. fumigatus* and *C. albicans* were evaluated in order to mimic secondary infections in critically ill COVID-19-infected patients. In comparison to healthy controls, these patients had an immune phenotype considered increased in HLA-DR^+^CD38^+^ and PD-1^+^ CD4^+^ and CD8^+^ T cells, with high CD8^+^CD244^+^ lymphocytes. Some monocyte activation markers—IL-6, IL-8, TNF, IL-10, and sIL2R*α*—were increased; however, IL-1β levels were low. Moreover, *A. fumigatus* antigen stimulation triggered an immune response, with no difference between COVID-19 patients and healthy controls, but a reduced monocyte CD80 upregulation. Regarding *C. albicans* responses, there was a lower release of IL-6, TNF, IL-1α, and IL-1β, and it was concluded that COVID-19 cases are more susceptible to *Candida* spp. infections [10]. Despite the marked immune dysregulation in COVID-19, no prominent defects have been reported in immune cells that are critically required for immunity to *Candida* spp. [6].

Among secondary infections in COVID-19 patients, APA showed to be related to a high rate of mortality and morbidity in patients with severe pneumonia. However, many features of the disease indicate that there are several diagnostic and therapeutic challenges that still need to be uncovered, since some cases with CAPA are undiagnosed through the lack of clinical awareness and global emergence of triazole resistance [122]. Moreover, a proper host immune response not only can protect against coronavirus, but may also restore immune hemostasis to reduce the risk of CAPA in COVID-19 patients [123]. The inflammatory cytokine cascade impairs the lung epithelial cells by producing large amounts of cytokine IL1α, which results in the production of IL1β from activated neutrophils and monocytes. Furthermore, the innate system also produces an extra level of nucleotide-binding leucine-rich repeat-containing proteins, or NOD-like receptors (NLRs), especially NLRP3 inflammasome, subsequently enhancing the level of IL6 and triggering detrimental responses associated with cytokine cascade [124]. In some cases of patients with aspergillosis, an increased level of IL6 is noticed (in epithelial cells), suggesting that a co-infection of COVID-19 may contribute to the severity of this clinical feature, owing to the augmented level of cytokines [125]. In this regard, in a large population of COVID-19 patients, the use of l IL6 receptor antagonist was stated to stimulate and sustain the immune response related to clinical development [126]. In contrast, trials in animal models with IL6 deficiency showed an association of this deficiency and a predisposition to CAPA [127], and thus, both IL6 receptor antagonist and antifungal are considered as prophylaxis in severe COVID-19 patients.

While there is much to be learned, our current understanding regarding co-infections in COVID-19 patients has provided some alternative immunotherapeutic strategies. This includes endogenous pathways of immunomodulation, which are recognized as a way to re-equilibrate the immune system, to overwhelm its complexity in COVID-19, and to prevent secondary infections, particularly aspergillosis [128]. For instance, thymosin α1, an endogenous thymic peptide with a wide range of immunomodulatory activities, could have beneficial effects on the activation of the immune system, and on balancing impaired immune responses, also inducing the indoleamine 2,3-dioxygenase 1 pathway [129,130,131]. Surprisingly, thymosin α1 effectively induced the antifungal activity, through the promotion of IFN and Th1 responses. Accordingly, it stimulates such responses in cases with active COVID-19 infection, but has no protective effects when used in prophylaxis [132,133,134]. Moreover, thymosin α1 could enhance immunomodulatory responses to vaccine and, subsequently, reduce COVID-19-associated secondary infections, specifically in elderly people [133,134]. Collectively, the normalization of immune responses might be an effective way of fighting aspergillosis. In controversy, it is arguable that Anakinra, a recombinant version of IL-1 receptor antagonist [135], could also restore immune responses for protection against aspergillosis in COVID-19 patients [136]. This drug has a favorable safety profile, and its efficacy against aspergillosis has been established as a result of unbalanced inflammasome activation in cystic fibrosis patients [137] and chronic granulomatous disease CGD, which leads to susceptibility to aspergillosis [138]. Likewise, there is a controversial idea on the protective role of aryl hydrocarbon receptor (AhR), a xenobiotic receptor, in COVID-19 patients susceptible to aspergillosis. Still, its beneficial therapeutic effects were linked to a reduction in the mucosal damage and re-establishment of the protection against gut infection, by stimulation production of IL-12. Hence, more studies will be required to assess the therapeutic purpose of AhR [139,140,141]. Recently, a study indicated that intravenous immunoglobulin (IVIg), collected from recovered patients (especially at the same geographic area), decreases inflammation of intestinal epithelial cells in newly infected subjects, and eradicates overgrowth of *C. albicans* in murine gut [142].

The application of effective natural compounds enhancing the capacity of the immune system are also drawing attention. Indeed, there are new insights into promising agents that can reduce the risk of infectious disease, specifically fungal pathogens, in susceptible individuals with COVID-19. Among them, honey and its ingredients showed a potential benefit towards inflammation disease and microbial pathogens such as fungal agents; however, further studies are needed on the application of honey [143]. In addition, β-glucan, a natural immunomodulatory component derived from *Saccharomyces cerevisiae*, was suggested to bolster innate immune responses in COVID-19 patients prior to infection, and any microbial infection as prophylaxis [144]. However, clinical trials are still needed to confirm its efficacy and to further study the distinctive effects of β-glucans from different sources.

## 4. Antifungal Resistance and Therapeutic Approaches in COVID-19 Patients

In recent years, we have been witnessing an incredible number of emerging resistant species related to a higher morbidity and mortality rates [145]. It has been estimated that, in 2050, antimicrobial resistance (AMR) could be responsible for 10 million deaths and treatment costs as high as USD 100 trillion [145]. This is also relevant in fungal infections. Indeed, the antifungal resistance phenomenon is especially critical in emerging resistant species, such as *C. auris* [17].

As seen during hospitalization, patients with COVID-19 are more predisposed to co-infections with bacterial and/or fungal pathogens (e.g., *C. albicans* and *A. flavus* [146,147]), which is likely to influence mortality rates [148,149,150]. Zhou et al. reported that almost 50% of mortalities accrued in patients had secondary bacterial and fungal infections [151]. This is the reason why antibiotics have been prescribed for hospitalized patients, for example, as a prophylactic measure against secondary infections, regardless of the susceptibility of the microorganism, promoting the emergence of multiple drug-resistant microbial species [3].

Since the onset of the COVID-19 pandemic, there are still few data on the prevalence of co-infections in patients with COVID-19 pneumonia. Yet, some studies already mention the problem of co-infections and drug resistance, which is the case of *Candida* spp. and COVID-19-associated superinfection mycosis, and its high potential for antifungal resistance [152]. Indeed, around 21% of patients who were under treatment with antifungals (voriconazole, isavuconazole, and caspofungin) showed no survival benefit [153]. Arastehfar et al. described COVID-19-associated candidemia (CAC) among seven Iranian patients. Half of patients with *C. albicans* were refractory to both azoles and echinocandins. Despite antifungal therapy, the high mortality of patients with CAC unveiled the severity of the disease in these patients. This, of course, also draws attention of the underestimation of the importance of an early diagnosis and timely initiation of antifungal therapy [121]. Another case reported a patient with COVID-19 CAPA caused by a triazole-resistant *A. fumigatus*, which highlights the need for surveillance triazole-resistant fungal species, particularly in CAPA cases [154]. Furthermore, early screening for IA and the necessity to identify isolates for pan-azole resistance should be considered in respiratory specimen in COVID-19 CAPA in ICU hospitalized patients [155].

Regarding antifungal susceptibility pattern of oropharyngeal candidiasis (OPC), a study carried out in Iranian COVID-19 patients showed that the majority of the *Candida* isolates were susceptible to all three classes of antifungal drugs (azoles, polyenes, and echinocandins). The only exception was one isolate of *Pichia kudriavzevii* and *C. dubliniensis*, which were caspofungin-resistant [156]. Long-term use of azoles may result in the selection of less sensitive species, such as *P. kudriavzevii*, *C. dubliniensis*, and *C. glabrata*, and in the development of drug resistance, even in previously susceptible *Candida* spp. [157]. Further studies should be carried out to design appropriate prophylaxis strategies in OPC.

Likewise, *C. glabrata* was recently linked to a possible fatal blood stream infection in a type-2 diabetes patient diagnosed with COVID-19. After 13 days of caspofungin treatment, *C. glabrata* with FKS-associated pan-echinocandin resistance was isolated from the patient [39]. Similarly, *C. auris* has been recovered from two-thirds of 15 cases of candidemia in India, with a high rate of fatality (60%). All *C. auris* isolates were resistant to fluconazole, and 40% were resistant to amphotericin B. This resistance to both classes of drug is highly concerning, because the use of other antifungals (such as echinocandins) are limited in developing countries [17]. In resource-limited countries, *C. auris* diagnostics are still a challenge, alerting the global medical community about the potential of *C. auris* as a critical factor in COVID-19 patients [158]. This also emphasizes the importance of early diagnosis and screening for antimicrobial drug-resistant co-infections, to reduce unfavorable outcomes in COVID-19 patients.

Commercial antibiotics and antifungals used for treatment of infectious diseases are almost all cytotoxic in high doses, which limits the use of these synthetic drugs. In this context, novel antimicrobial agents are among the most popular therapeutic strategies currently being applied, with minimal side effects to reduce AMR. New drug delivery systems including nano-carriers, liposomes, nano-mesopores, and nano carbon tubes plus the natural or bioactive compounds are promising therapeutic agents that have recently interested researchers [159,160]. In addition, many essentials oils, plant extracts, and essences have also been investigated and the results showed that these compounds have potential antibacterial, antifungal, and antiviral specification [161,162,163]. Still, further investigations are required to prove their activity in the future.

## 5. Role of the Microbiome and Probiotics to Fight COVID-19

At the beginning of the COVID-19 pandemic, several studies confirmed the prominent role of the immune system to defeat pathogens in COVID-19 cases [164]. Numerous clinical and scientific studies instill a promising window, considering that the gastrointestinal (GI) tract has a fundamental role to enhance the host immunity of COVID-19 [149,165]. In this regard, (normal) microbiota are described as the population of microorganisms (e.g., fungi, virus, bacteria) which particularly exist in the gut, with beneficial activity for the host (e.g., production of vitamins, facilitation of digestion, and stimulation of immune response against pathogens) [1,166,167,168]. Therefore, physiological changes in the intestinal tract easily lead to infection and inflammation disorders.

SARS-CoV-2 induces infections through binding angiotensin-converting enzyme 2 (ACE2) receptor, which is expressed on the cell surface of esophagus, lung, liver, and intestinal epithelium [169,170,171]. The microbiota that colonize the epithelial membrane of skin, oral cavity, and gut play an essential role to boost immunity in targeted tissues to fight and inhibit the adhesion of several pathogens [166]. In addition, during the fermentation process, the metabolites produced by microbiota can inhibit ACE2 receptors and suppress the implantation of viruses. As a result, blocking ACE2 receptor or blocking viral proteins could avoid any development of this viral infection [172,173,174].

Previous reports demonstrated that, in healthy cases, the primary community of microbiota in the oral cavity includes *Firmicutes*, *Bacteroidetes*, *Proteobacteria*, *Actinobacteria*, *Spirochaetes*, and *Fusobacteria*, while the major fungal species includes *Candida* spp., followed by *Cladosporium* spp., *Aureobasidium* spp., and Saccharomycetales [175,176]. The diversity of microbiota changes with age, since the population of microbiota in infants is less than adults, and in elders, is less than young people, which supports the evidence that the elderly are more susceptible to COVID-19 infection [177]. Besides, the gut microbiota also regulate the intestine mucosal site through the production of metabolites such as short chain fatty acids (SCFs), which can restore the secretion of immunoglobulins, effector cells, and anti-inflammatory factors (e.g., NF-kβ and TNF-α) in healthy individuals via affected pattern recognition receptors (PRR) [171]. On the other hand, microbiota metabolites binding with toll-like receptors (TLR) consequently regulate the immune responses and increased expression of T regulatory lymphocytes, cytokines, and chemokine to inhibit viral infection. It has been indicated that a direct relationship exists between microbiota and COVID-19 infection. *Bacillus subtilis* reduced the infectivity of COVID-19 [174]. Moreover, in COVID-19 patients’ lungs, the microbiota compounds were altered. The changes were thought to have an essential role in the COVID-19 immunity, severity of clinical presentation, and outcome [178].

As microbiota can affect antiviral immunity, probiotics are indicated as having anti-viral, anti-inflammatory, and anti-allergic activities, and to decrease the time duration and rate of respiratory viral infections [167,168,179,180]. Commensal fungi are well-known mycobiota, which directly and indirectly impact virus pathogenesis in the lungs [173]. The main fungal population in healthy cases include *Candida* spp., followed by *Cladosporium* spp., Saccharomysetales, and *Aureobasidium* spp. [1]. Based on previous studies, the mycobiome is significantly altered in COVID-19 patients when compared with healthy subjects [181,182]. Indeed, probiotic microorganisms, including *Saccharomyces* spp., *Lactobacillus* spp. and *Bifidobacterium* spp., are broadly used in the food industry, having important functions in innate immune response and modulating of immune cells such as B and T lymphocytes, macrophages, and dendritic cells [183]. The possible mechanism of probiotic immune modulation includes the activation of TLRs [184], regulation of gene expression and signaling pathways in the host cells [159,185]. It has also been disclosed that bacteria metabolites regulate the mucosal immunity via interacting with TLRs, cytokines, chemokines, and expression of NF-kB. In fact, it is known that several signals received from the lower GI tract can be transmitted to other mucosal surfaces, such as the respiratory tract, thereby enhancing protection against infection [171]. Although lungs possess their own microbiota, the inhibition of viral replication by lung–gut microbiota interaction indirectly influences the immune response of the respiratory tract [186,187]. This interaction occurs as host–microbe or microbe–microbe and affects the course of the respiratory infection. Importantly, any imbalance in communities of lung–gut microbiomes (dysbiosis) has been related to severe respiratory infections [187,188]. Intestinal dysbiosis was indicated to cause inflammation and weaker response to pathogens [189]. Even though our knowledge about fungal microbiota and probiotics is restricted, it is, for example, supported that supplementation with the combination of *Streptococcus thermophilus* and *Bifidobacterium bifidum* promote the reduction of viral infections [190]. The oral administration of *Lactobacillus acidophilus* in mice indicated a decrease in inflammation and damage on lung tissues after 24 h of a pulmonary infection. The diversity of the microbiome in a population creates a different range of severity of infections in each individual. The use of probiotics might open a new insight into the management of fungal pathogens, but there is much to uncover about the probable side effectiveness of clinical application of those in COVID-19 cases [191,192,193].

## 6. Final Remarks

Opportunistic fungal infections are of concern in COVID-19 patients. Categorically, these patients can develop fungal infections throughout any stages of this disease [23]. At the beginning, COVID-19 was highly associated with pulmonary aspergillosis and candidemia (invasive candidiasis), which were increasingly recognized as the main fungal diseases. Conversely, in recent months, a pointedly growing shift to other fungal infections has been ongoing. This is the case of infections related to *Mucor* and *Rhizopus* genera, *Cryptococcus* spp. and other less common species.

In general, data show that COVID-19 patients in ICU seem are more susceptible to fungal infections, when compared with patients without ICU admission, due to their immunosuppression status (the same case of HIV patients). Moreover, probably the incidence of aspergillosis is higher in COVID-19 patients, as the virus particularly affects the respiratory system. Correspondingly, in COVID-19 patients, the mortality rate is high in the case of co-infections (both bacterial and fungal species).

A highly complex interplay of predisposing factors, such as previous respiratory pathology, diabetes, nosocomial infection sources, and immunosuppressive therapy, is linked to co-infections. Furthermore, the neglected attitude towards fungal (tropical) diseases over the years, and the financial support for their diagnosis, treatment, and research, which is much lower than those available for other infectious diseases, leads to a similar mortality percentage [194]. Moreover, as COVID-19 patients are under immunosuppressive conditions, particularly T CD4^+^ and CD8^+^ lymphocytopenia, this provides an encouraging background for the occurrence of persistent fungal co-infections [116,117,118]. It is quite evident that any systemic immune alterations or the use of steroids to control COVID-19 may be directly related to the suppression in immunity, which also allows the colonization of opportunistic fungi. Hence, there is an urgent need to use steroids judiciously, prepare more comprehensive guidelines, and improve the steroid characterization for their efficacy, types, dose, duration of therapy, route of administration, and interaction with other drugs in order to improve COVID-19 treatments and prevent the increased probability and risk of developing a fungal infection secondary to the disease.

Investing more in precise guidelines related to the correct administration of antifungal agents and promoting more effective doses to increase the success of antifungal treatments is also imperative. Based on WHO guidelines for the control of resistant species, antimicrobial treatment/prophylaxis must be restricted, except when undertaking clinical indication [195]. This, obviously, also draws attention to the underestimation of the importance of an early diagnosis and timely initiation of antifungal therapy [121]. Of course, the adoption of certain precautions is also essential, such as hand washing and disinfecting surfaces with antiseptic agents.

Lastly, it also needs to be highlighted that several factors, such as lack of appropriate equipment to early screen and identify fungal infections, can result in many cases remaining undiagnosed. Subsequently, when the efficient treatment is not achieved on time and the multidrug-resistant phenomenon persists, this results in clinical failure outcomes in COVID-19 patients [196].

## Figures and Tables

**Table 1:** Clinical characteristics of COVID-19 patients reported with candidiasis.

Fungal Infection in COVID-19 Infection	Observed Immune Response	Co-Morbidity/ Disease Models	Test/Diagnosis Performed	COVID-19 Treatment	Antifungals Used	Steroids?	Outcome after Treatment	References
Candidemia *Candida duobushaemulonii* *Candida parapsilosis*, *Candida lusitaniae*	Elevated pro-inflammatory markers (d-dimer, ferritin, CRP, progressive thrombocytosis) and neutrophilia	Acute pulmonary embolism with subarachnoid hemorrhage superimposed bacterial pneumonia	CT scan, Culture, RT-PCR Blood, urine, and DTA	Meropenem, Levofloxacin Trimethoprim/sulfamethoxazole, Amikacin, tigecycline, colistin	Intravenous fluconazole	NR	Dead	[32]
Candidemia *(Candida glabrata)*	Leucocytes—normal, C-reactive protein and interleukin 6—altered	Type-2 diabetes ischemic heart disease stadium IV, leg amputation highly suspected bacterial superinfection	Chest X-ray and CT scan, RT-PCR, serology, MALDI-TOF	Darunavir/ritonavir, HCQ, piperacillin/tazobactam, teicoplanin, ertapenem, colistin	Caspofungin	NR	Dead	[39]
Candidemia *Candida auris* (n = 10), *Candida albicans* (n = 3), *Candida tropicalis* (n = 1), *Candida krusei (P. kudriavzevii)* (n = 1)	NA	Underlying chronic conditions (e.g., hypertension, n = 7; DM, n = 6; and chronic kidney and liver disease, n = 2)	MALDI-TOF and molecular identification—sequencing	NR	Micafungin	NR	Dead (n = 8)	[4]
Candidemia *Candida auris* (n = 3)	NA	DM, hypertension, chronic renal failure, coronary artery disease, obesity	Vitek 2 system, MALDI-TOF, sequencing, multiplex PCR	NR	Anidulafungin	NR	Dead	[36]
Candidemia *Candida auris* (n = 12)	NA	DM (n = 6), hypertension (n = 6), multiple myeloma (n = 1), stem cell transplantation (n = 1), dyslipidemia (n = 1), end stage renal disease (n = 1), bladder cancer (n = 1), obesity (n = 1), systematic lupus erythematosus (n = 1)	PCR, MALDI-TOF, Vitek2, whole genome sequencing	Remdesivir (n = 9), HCQ (n = 1),	Amphotericin B Micafungin,	n = 10	Dead (n = 6) Alive (n = 6)	[40]

DM: diabetes mellitus; DTA: deep tracheal aspirate; HCQ: Hydroxychloroquine; MALDI-TOF: matrix-assisted laser desorption/ionization time-of-flight; NA: not applicable/available; NR: not reported; PCR: polymerase chain reaction.

**Table 2:** Clinical characteristics of COVID-19 patients reported with aspergillosis.

Fungal Infection in COVID-19 Infection	Observed Immune Response	Co-morbidity/ DiseaseModels	Test/Diagnosis Performed	COVID-19 Treatment	Antifungals Used	Steroids?	Outcome after Treatment	References
Aspergillosis *Aspergillus* spp., CAPA	Highly permissive inflammatory response	DM, CVD	CT scan, Culture	HCQ	Azoles, liposomal amphotericin B	NR	Alive	[43]
	Immunocompromised	ARD, HT	CT scan, RT-PCR, Culture, ELISA	NR	Voriconazole	Yes (n = 7)	Some alive and some dead	[44]
Aspergillosis *Aspergillus fumigatus, CAPA*	Immunocompromised	DM, HT	CT scan, Culture	NR	Isavuconazole, voriconazole	No	Alive	[42]
		HT, coronary heart disease, obesity	CT scan, RT-PCR, Culture,	HCQ, meropenem, azithromycin	Voriconazole	Yes	Dead	[26]
	Low B-cell and T-cell response	Severe dyspnea, hypertension, DM	CT scan, RT-PCR, Serology	RD, multiple antibiotics	Multiple antifungals	No	Alive	[45]
	Systemic pro-inflammatory cytokine responses	Asthma, DM, Myeloma	CT scan, RT-PCR, Culture,	NR	Voriconazole, isavuconazole, liposomal amphotericin B, caspofungin, anidulafungin	Yes	Some alive and some dead	[46]
	High inflammatory response and immunosuppression	ALL, AML	RT-PCR, CT scan, Culture, Serology	NR	Caspofungin, fluconazole, liposomal amphotericin B, caspofungin, itraconazole	No	Some alive and some dead	[47]
Aspergillosis *Aspergillus* spp., IA	Acquired immunodeficiency and immunosuppression	ARD	Antigen, CT scan, Culture, Serology	NR	NR	Yes	Death (quick evolution)	[48]
	Strong deregulation of core components of innate immune and inflammatory responses	RHAEM	NA	NA	NA	NA	NR	[49]

ARD: acute respiratory disease/distress; ALL: acute lymphoblastic leukemia; AML: acute myeloid leukemia; CAPA: COVID-19-associated pulmonary aspergillosis; CT: computed tomography; CVD: cardiovascular disorder; ELISA: enzyme-linked immunosorbent assay; DM: diabetes mellitus; HIV: human immunodeficiency viruses; HT: hypertension; IA: invasive aspergillosis; NA: not applicable/available; NR: not reported; RHAEM: Reconstituted Human Airway Epithelial Model; RA: Rheumatoid arthritis; HCQ: Hydroxychloroquine; RD: Remdesivir; RT-PCR: real time-polymerase chain reaction.

**Table 3:** Clinical characteristics of COVID-19 patients reported with histoplasmosis.

Fungal Infection in COVID-19 Infection	Observed Immune Response	Co-morbidity/ Disease Models	Test/Diagnosis Performed	COVID-19 Treatment	Antifungals Used	Steroids?	Outcome after Treatment	References
Histoplasmosis *Histoplasma capsulatum*	Acquired immunodeficiency	HIV	CT-scan, RT-PCR	Tenofovir/lamivudine and atazanavir/ritonavir ceftriaxone, azithromycin	Itraconazole	Yes (dexamethasone)	Alive	[27,52]
	HIV	HIV	CT-scan, RT-PCR	Atazanavir/ritonavir, tenofovir/emtricitabine	Itraconazole, amphotericin B deoxycholate	No	Alive	[27]
	Inflammatory response	NA	CT-scan, RT-PCR	Levofloxacin	Itraconazole	Yes (methylprednisolone)	Alive	[53]
	NA	NA	CT scan, RT-PCR	NA	Itraconazole	No	Alive	[54]
Histoplasmosis *Histoplasma capsulatum*-like intracellular yeasts	Acquired immunodeficiency	HIV	CT-scan, RT-PCR	HCQ, lopinavir/ritonavir, tenofovir disoproxil fumarate/emtricitabine plus dolutegravir	Amphotericin B deoxycholate, itraconazole	No	Lost to follow-up	[55]

ART: antiretroviral therapy; CT: computed tomography; HIV: human immunodeficiency viruses; NA: not applicable/available; HCQ: Hydroxychloroquine; RT-PCR: real time-polymerase chain reaction.

**Table 4:** Clinical characteristics of COVID-19 patients reported with mucormycosis.

Co-Morbidity/ DiseaseModels	Test/Diagnosis Performed	COVID-19 Treatment	Antifungals Used	Steroids?	Outcome after Treatment	References
Obesity HT	CT-scan, RT-PCR	None mentioned	Linezolid, meropenem	NA	Died	[67]
Asthma HT DM	CT-scan, RT-PCR	Remdesivir	Amphotericin B	NA	Died	[68]
DM Vascular disease	CT-scan, RT-PCR	Tocilizumab, methylprednisolone, dexamethasone	Amphotericin B	Methylprednisolone, dexamethasone	Died	[69]
HT	CT-scan, RT-PCR	Hydrocortisone	Amphotericin B	Hydrocortisone	Died	[70]
NA	CT-scan, RT-PCR	Remdesivir, tocilizumad, dexamethasone	Amphotericin B	Dexamethasone	Died	[71]
Asthma HT DM	CT-scan, RT-PCR	Remdesivir, dexamethasone	Amphotericin B	Dexamethasone	Died	[72]
HT	CT-scan, RT-PCR	HCQ, lopinavir–ritonavir	Amphotericin B	NA	Died	[73]
DM ICM RD	CT-scan, RT-PCR	Meropenem	Amphotericin B	Dexamethasone	Alive	[74]
DM	CT-scan, RT-PCR	NA	Amphotericin B	NA	Alive	[75]
HT, DM	CT-scan, RT-PCR	NA	Liposomal amphotericin B, itraconazole	NA	Alive	[76]
NA	RT-PCR CT-scan	Remdesivir, dexamethasone, metformin, glipizide	Amphotericin B, ceftriaxone	Dexamethasone	Live	[77]
DM	CT-scan, RT-PCR	Meropenem, oseltamivir tocilizumab, sitagliptin/metformin	Amphotericin B	Methylprednisolone, dexamethasone	Died	[69]
DM	CT-scan, RT-PCR	Remdesivir, ceftriaxone, azithromycin, dexamethasone	Voriconazole, liposomal amphotericin B	Dexamethasone	Live	[78]
DM (1 patient) No co-morbidity (1 patient)	CT-scan	Remdesivir, convalescent plasma, vancomycin, piperacillin-tazobactam	Amphotericin B	NA	Live (n = 1) Died n = (1)	[68]
Obesity DM	CT-scan, RT-PCR	Amoxicillin-clavulanate, imipenem/linezolid	Amphotericin B	NA	Died	[79]
DM (n = 8) CRF (n = 3)	CT-scan	Broad-spectrum antibiotics	Liposomal amphotericin B	Dexamethasone	Died (n = 7) Alive (n = 4)	[80]
DM HT (all patients)	RT-PCR	HCQ, glucocorticoids	Systemic antifungals	Glucocorticoids	Died (n = 7) Live (n = 8)	[81]
T2DM (4) T2DM with HT (1) HT (1) Kidney Disease (1)	CT-scan, RT-PCR	Tocilizumab, prednisolone, piperacillin/tazobac, linezolid	Voriconazole	Prednisolone	Died (n = 3) Alive (n = 4)	[82]
DM (21-cases) HT (14-cases) Renal failure (1-case)	CT-scan, RT-PCR	HCQ, azithromycin	Caspofungin	Combination of steroids	All Live	[76]
DM (16)	RT-PCR	Corticosteroids	Liposomal amphotericin B, voriconazole, posaconazole	On Steroid	Alive (n = 10) Died n = (6)	[83]
HT, UTI	CT-scan, RT-PCR	Either dexamethasone or methylprednisolone (7 patients); interferon (2 patient); remdesivir (1 patient); flavipiravir and HCQ (1 patient)	Amphotericin B, posaconazole	Dexamethasone or Methylprednisolone (n = 7)	Live	[84]
DM	RT-PCR CT-scan	Remdesivir, levofloxacin, dexamethasone, meropenem, vancomycin, piperacillin/tazobactam	Amphotericin B, posaconazole	Dexamethasone	Live	[85]
No co-morbidity	CT-scan, RT-PCR	HCQ	Amphotericin B	NA	Died	[86]
chronic lymphocytic leukemia DM	RT-PCR	NA	Amphotericin B	NA	Died	[87]
DM HT asthma	RT-PCR	NA	Amphotericin B	No	Died	[88]
AML	CT-scan, RT-PCR	HCQ lopinavir-ritonavir	Amphotericin B	NA	Died	[73]
renal disease	CT-scan, RT-PCR	Remdesivir, vancomycin, cefepime	Liposomal amphotericin B, posaconazole	Dexamethasone	Died	[72]
ICM HF s/p OHT DM HT CKD	RT-PCR	Remdesivir methylprednisolone	Fluconazole	Methylprednisolone, dexamethasone	Died	[89]
No history of any co-morbidity	CT-scan, RT-PCR	Tocilizumab	Liposomal amphotericin B, posaconazole, isavuconazole	Dexamethasone	Live	[90]
DM HT		Piperacillin/tazobactam, HCQ, azithromycinlopin, vir/ritonavir, prednisone Dexamethasone	Liposomal amphotericin B, isavuconazole, posaconazole	Prednisone, Dexamethasone	Live	[91]
HT	RT-PCR	Remdesivir, dexamethasone	Amphotericin B	Dexamethasone	Died	[92]
T2DM (all 6 patients)	CT-scan, RT-PCR	Prednisolone, dexamethasone, methylprednisolone	Amphotericin B, posaconazole	Prednisolone, Dexamethasone, methylprednisolone	All Live	[93]
DM HT	CT-scan, RT-PCR	Remdesivir, interferon-alpha	Systemic antifungals	Systemic corticosteroid	Died	[94]
T2DM, HT (2) T2DM (3)	CT-scan, RT-PCR	Tocilizumab, convalescent plasma, methylprednisolone	Liposomal amphotericin B, posaconazole	Methylprednisolone	Died (n = 2) Alive (n = 3)	[95]
T1DM	CT-scan, RT-PCR	Ceftriaxone, azithromycin, dexamethasone, remdesivir, tocilizumab	Amphotericin B	Dexamethasone	Live	[71]
Obesity hypothyroidism	CT-scan, RT-PCR	HCQ, remdesivir, vancomycin, meropenem	Liposomal amphotericin B, posaconazole	Prednisone	Died	[96]
HT Asthma	RT-PCR	Meropenem, remdesivir, dexamethasone	Liposomal amphotericin B	Dexamethasone, prednisolone	Died	[97]

CT: computed tomography; DM: diabetes mellitus; HIV: human immunodeficiency viruses, HT: hypertension; NA: not applicable/available; HCQ: Hydroxychloroquine; RT-PCR: real time-polymerase chain reaction; ICM: ischemic cardiomyopathy.; CKD: chronic kidney disease; AML: acute myeloid leukemia; UTI: urinary tract infections: HF; heart failure; s/p: status post; OHT: orthotopic heart transplant.

**Table 5:** Clinical characteristics of COVID-19 patients reported with cryptococcosis and other fungal infections.

Fungal Infection in COVID-19 Infection	Observed Immune Response	Co-morbidity/ Disease Models	Test/Diagnosis Performed	COVID-19 Treatment	Antifungals Used	Steroids?	Outcome after Treatment	References
*Cryptococcus neoformans*	High inflammatory response and immunosuppression	HAT, HBV	CT-scan, RT-PCR	meropenem, vancomycin	Fluconazole	Yes (tacrolimus, prednisone)	Death	[102]
	Acquired immunodeficiency and immunosuppression	HIV	CT-scan, RT-PCR	Tenofovir-DF/ Emtricitabine-atazanavir/ritonavir	Amphotericin B deoxycholate plus fluconazole	No	Death	[103]
	High inflammatory response and immunosuppression	Stage IV prostate cancer HT, colon-sigma diverticulosis	CT-scan	No	Fluconazole Amphotericin B plus flucytosine	Dexamethasone	Death	[104]
	High inflammatory response and immunosuppression	HT, DM	NA but COVID19 positive mentioned	Tocilizumab and corticosteroids	Anidulafungin, Amphotericin, flucytosine	Methylprednisolone	Death	[98]
Coccidioidomycosis (*Coccidioides immitis, C. posadasii*)	Impaired cytokine signaling from CD4+ Th1 and cytotoxic CD8+ T-cells among patients	No associated respiratory symptoms & disease	CT scan, Culture, Serology	NR	Liposomal Amphotericin B	No	Alive	[105]
Coccidioidomycosis (*Coccidioides immitis)*	Depressed cellular immunity	Progressive respiratory symptoms, hypoxemia	CT scan, Culture,	Remdesivir	Fluconazole	No	Alive	[106]
*Pneumocystis jirovecii*	Cytokine release storm	RA	CT scan, Culture, Serology	HCQ, Tocilizumab	Caspofungin, ganciclovir, cefoperazone	Glucocorticoids	NR	[107]
	Functional immune suppression related to CD4^+^ lymphocytopenia	HIV, progressive hypoxemia	RT-PCR, Culture, Serology, CT	NR	Trimethoprim- sulfamethoxazole	NR	NR	[108]
	Immunocompromised	ARD, DM, HT	RT-PCR, Culture, Serology,	HCQ, Lopinavir-ritonavir	Antifungals and antibacterials	Yes	Some alive and some dead	[109]
	Low CD4 count (35.6%)	HIV	CT, RT-PCR, Multiplex PCR	NR	Co-trimoxazole and oral prednisolone	No	Alive	[110]
	Anemia, lymphopenia, raised C-reactive protein, immunosuppression	HIV	CT, RT-PCR	NR	Co-trimoxazole, IV pentamidine	No	Death	[111]
	Severe depletion of CD4^+^ cells	HIV	RT-PCR, Culture, CT	Emtricitabine, Ritonavir	Trimethoprim-sulfamethoxazole	No	NR	[99]
	Immunocompetent patient	Recovered from COVID-19	RT-PCR, Culture, CT	Enoxaparin, ceftaroline	Trimethoprim-sulfamethoxazole, methylprednisolone	Yes	Alive	[100]
	Immunocompromised patients	HT, hepatic steatosis, massive lung thromboses	RT-PCR, Culture, CT, Histopathology	Remdesivir	Trimethoprim-sulfamethoxazole, prednisone	Yes	Some alive and some dead	[101]
*Saccharomyces cerevisiae (boulardii)* (n = 2)	Immunosuppression	HT (first) Diabetes (Second)	RT-PCR	Oseltamivir HCQ	Anidulafungin, fluconazole	No treated with Ultra-Levure [preparation of *Saccharomyces cerevisiae* (*boulardii*)]	Both live	[112]
*Fusarium proliferatum*	immunocompetent diabetic patient	HAT substituted hypothyroidism	RT-PCR	No	Amphotericin B caspofungin	No	Live	[113]

ARD: Acute respiratory disease/distress, CT: Computed tomography, DM: Diabetes mellitus; HIV: human immunodeficiency viruses; HT: Hypertension; IA: Invasive aspergillosis; NA: Not applicable/available; NR: Not reported; RHAEM: Reconstituted Human Airway Epithelial Model; RA: Rheumatoid arthritis; HCQ: Hydroxychloroquine; RD: Remdesivir; RT-PCR: real time-polymerase chain reaction; HBV: hepatitis B virus.

## Data Availability

Not applicable.
